# A Case of Reactive Lymphoid Hyperplasia That Is Difficult to Differentiate From Renal Cancer With Lymph Node Metastasis

**DOI:** 10.1002/iju5.70162

**Published:** 2026-03-08

**Authors:** Shunki Nakagawa, Yasutomo Nakai, Yujiro Hayashi, Yutaka Kurahashi, Shu Okamoto, Yuichiro Nakamura, Norihiko Kawamura, Akira Nagahara, Masashi Nakayama, Kazuo Nishimura

**Affiliations:** ^1^ Department of Urology Osaka International Cancer Institute Osaka City Japan

**Keywords:** differential diagnosis, kidney, lymphadenopathy, reactive lymphoid hyperplasia, renal tumor

## Abstract

**Introduction:**

Reactive lymphoid hyperplasia (RLH) is a rare benign lymphoproliferative disorder that rarely involves the kidney and has not been reported in association with regional lymphadenopathy.

**Case Presentation:**

A 70‐year‐old woman was incidentally found to have a left renal mass. Contrast‐enhanced computed tomography revealed a 20‐mm enhancing renal lesion with enlarged hilar lymph nodes, leading to a preoperative diagnosis of renal cell carcinoma with nodal metastasis (cT1aN1M0). Open radical nephrectomy with regional lymphadenectomy was performed. Histopathological analyses established a diagnosis of reactive lymphoid hyperplasia. No additional treatment was administered, and the patient has remained free of recurrence for 3 months.

**Conclusion:**

We report the first case of RLH accompanied by regional lymph node enlargement. Because RLH can closely mimic renal cell carcinoma on imaging, it should be considered in the differential diagnosis of renal masses when the clinical presentation is inconsistent with typical renal cell carcinoma.

## Keynote Message

Renal reactive lymphoid hyperplasia is an extremely rare benign condition that can present with regional lymphadenopathy and closely mimic renal cell carcinoma on imaging. When the clinical presentation is inconsistent with typical renal cell carcinoma, percutaneous needle biopsy should be considered, provided it can be performed safely, to avoid unnecessary radical surgery.

## 1 Introduction

Reactive lymphoid hyperplasia (RLH) is a benign lymphoproliferative disorder characterized histologically by lymphoid follicles with reactive germinal centers and polyclonal lymphocytic proliferation without atypia [1]. RLH has been reported in several organs, including the liver [2], skin [3], gastrointestinal tract [4], and orbit [5], but its occurrence in the kidney is exceedingly rare. Moreover, no previously reported renal RLH cases have been associated with regional lymphadenopathy.

Because renal RLH can closely resemble renal cell carcinoma on imaging, accurate preoperative diagnosis is difficult. Here, we report a rare case of renal RLH initially suspected to be renal cell carcinoma with regional lymph node metastasis.

## 2 Case Presentation

**FIGURE 1:**
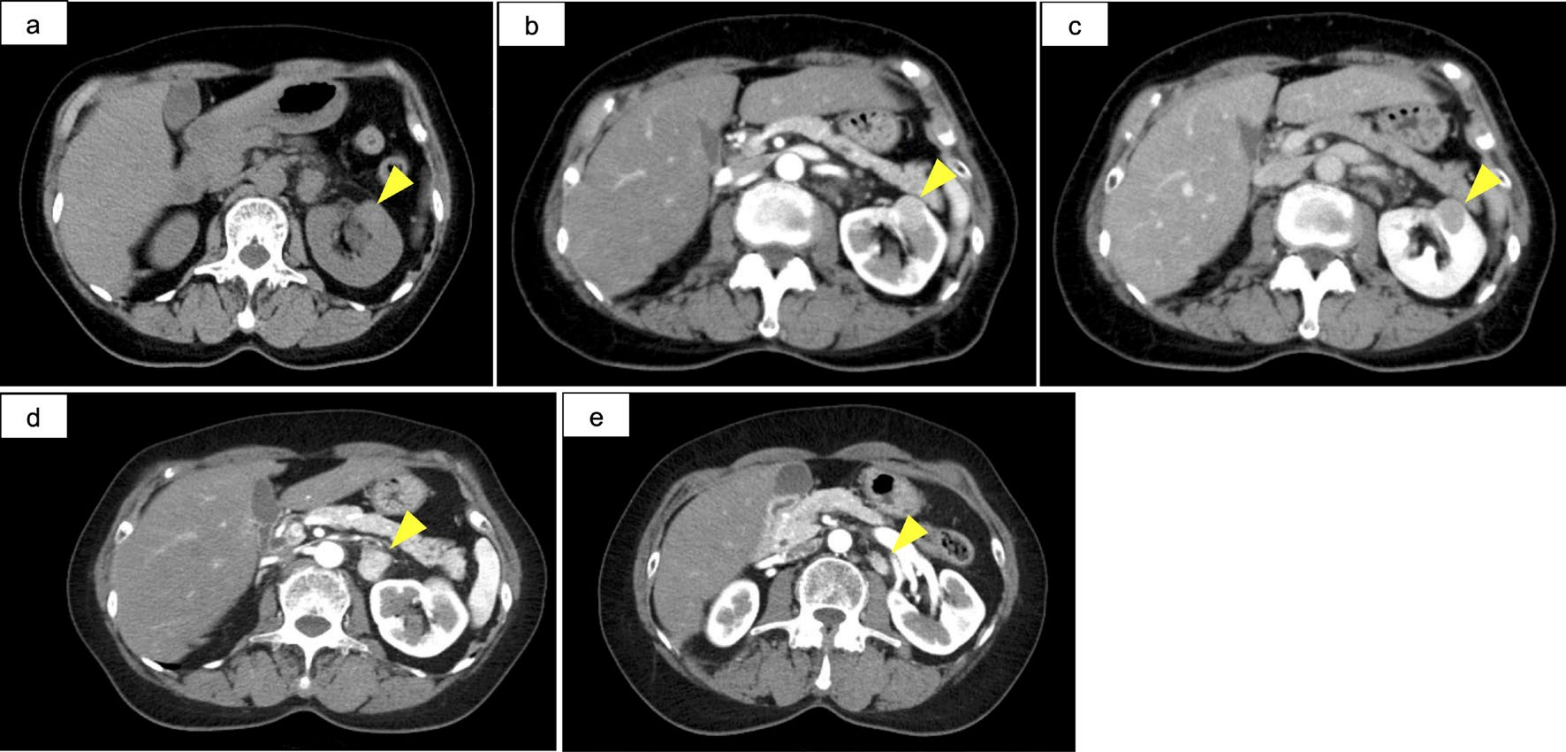
Preoperative contrast‐enhanced CT images. (a) Non‐contrast image. (b) Early‐phase contrast‐enhanced CT showing relatively homogeneous enhancement of the renal tumor. (c) Late‐phase contrast‐enhanced CT showing gradual washout of the renal tumor. (d, e) Early‐phase contrast‐enhanced CT showing two enlarged lymph nodes in the renal hilar region with early enhancement.

A 70‐year‐old woman with no significant symptoms was incidentally found to have a left renal mass during a routine health checkup. Contrast‐enhanced computed tomography (CT) revealed a 20‐mm renal lesion demonstrating early homogeneous enhancement with gradual washout, accompanied by two enlarged hilar lymph nodes showing a similar enhancement pattern (Figure 1). No evidence of distant metastases was identified.

Based on these imaging findings, renal cell carcinoma with lymph node metastasis (cT1aN1M0) was suspected, and the patient was referred to our department. Her Eastern Cooperative Oncology Group performance status was 0, and laboratory tests were unremarkable. Although the primary tumor was small, the presence of enlarged lymph nodes with suspicious enhancement led to a preoperative diagnosis of renal cell carcinoma with lymph node metastasis. Percutaneous needle biopsy of the renal tumor was not attempted due to the close proximity of the tumor to the pancreas. Consequently, open radical nephrectomy with lymph node dissection was planned.

### 2.1 Surgical Findings

**FIGURE 2:**
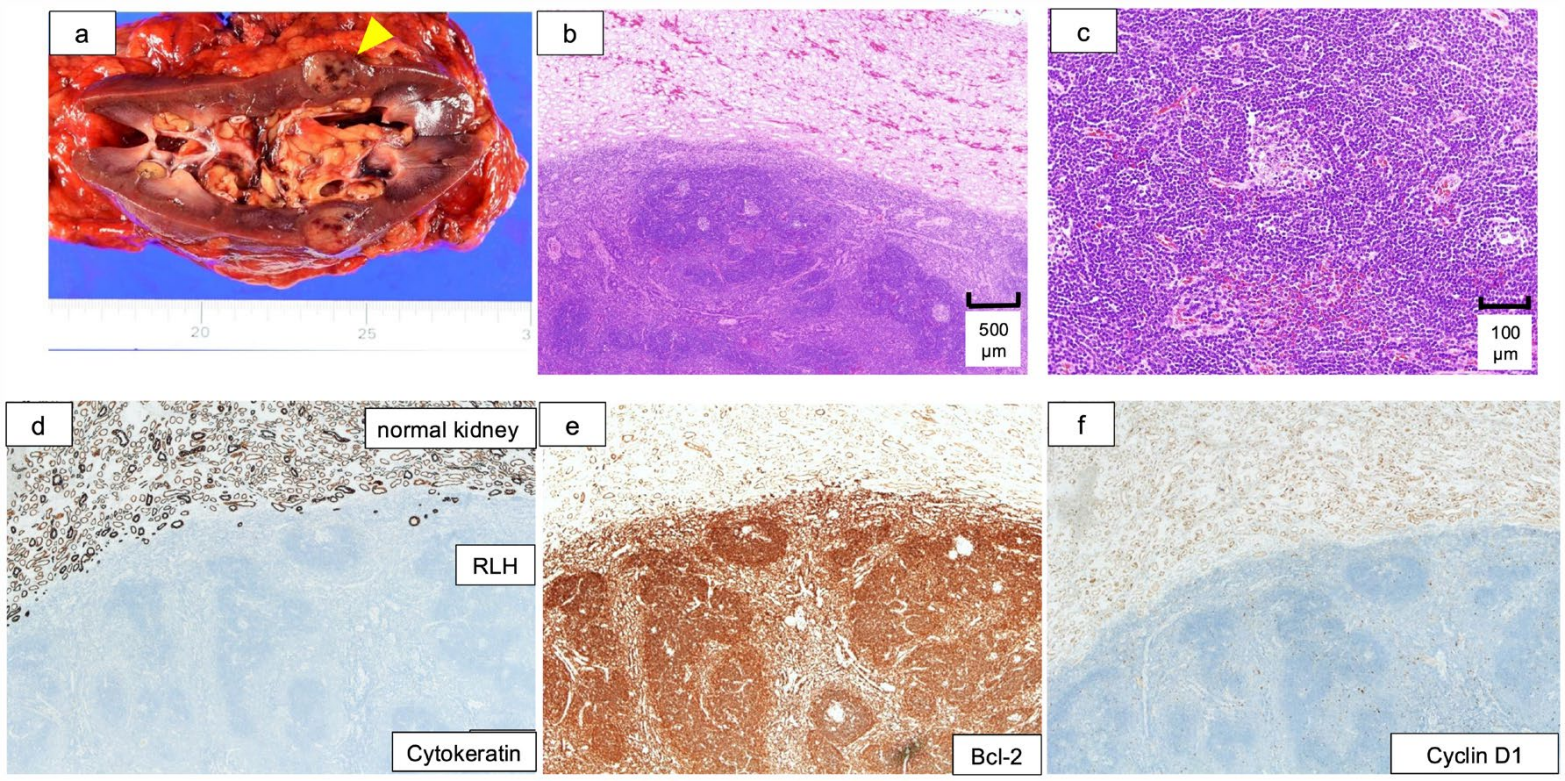
Nephrectomy specimen, hematoxylin–eosin (HE) staining, and immunohistochemistry. (a) Gross specimen showing the renal tumor and enlarged hilar lymph nodes resected en bloc. (b) HE staining, low magnification, revealing lymphoid follicular architecture. (c) HE staining, high magnification, showing reactive germinal centers. (d) Cytokeratin immunostaining: Negative in the lesion, excluding epithelial malignancy. (e) Bcl‐2 immunostaining: negative in germinal centers, ruling out follicular lymphoma. (f) Cyclin D1 immunostaining: negative, excluding mantle cell lymphoma.

Open radical nephrectomy with regional lymphadenectomy was performed via a transperitoneal approach. The renal tumor and enlarged hilar lymph nodes were resected en bloc. The total operative time was 4 h and 24 min, with estimated blood loss of 237 mL. The weight of resected specimen was 314 g (Figure 2a).

### 2.2 Pathological Findings

Histopathological examination of both the renal tumor and the enlarged lymph nodes revealed lymphoid tissue with minimal cytological atypia, characterized by reactive germinal centers and interfollicular lymphocytic aggregation (Figure 2b,c). Immunohistochemical staining for cytokeratin was negative in both lesions, thereby excluding epithelial malignancies such as renal cell carcinoma (Figure 2d). Further immunohistochemistry showed negative Bcl‐2 expression in germinal centers and absence of cyclin D1 expression, ruling out follicular lymphoma and mantle cell lymphoma, respectively (Figure 2e,f).

These findings confirmed the diagnosis of reactive lymphoid hyperplasia. No additional treatment was administered, and the patient has remained free of recurrence for three months.

## 3 Discussion

RLH is a benign lymphoproliferative disorder that rarely involves the kidney. In the differential diagnosis of renal RLH, IgG4‐related disease and low‐grade B‐cell lymphoma, particularly MALT lymphoma, should be carefully excluded. IgG4‐related disease is characterized by increased IgG4‐positive plasma cells and storiform fibrosis [6], whereas MALT lymphoma typically demonstrates monoclonality and lymphoepithelial lesions and may show cyclin D1 positivity in certain subtypes [7]. In the present case, the absence of cytologic atypia, negative Bcl‐2 staining in germinal centers, and lack of cyclin D1 expression supported a diagnosis of reactive lymphoid hyperplasia.

Only a limited number of cases of renal RLH have been reported, all of which presented as solitary masses without associated lymphadenopathy [8, 9, 10] (Table 1). Therefore, to the best of our knowledge, the present case is the first report of renal RLH accompanied by regional lymph node enlargement, closely mimicking renal cell carcinoma with nodal metastasis.

**TABLE 1:** Clinicopathological features of reactive lymphoid hyperplasia (RLH) of the kidney reported in the literature.

Author	Sex	Age	Size	Regional lymphadenopathy	Treatment	Associated disorders
Fukuda (6)	M	70	20 mm	None	Surgery	n.a.
Wada (7)	F	68	n.a.	None	Drugs (Prednisolone)	Pseudolymphoma of the orbit
Cacoub (8)	M	56	40 mm	n.a.	Drugs (Prednisolone)	Sjogren syndrome
Nakagawa	F	70	20 mm	21 mm, 10 mm	Surgery	n.a.

*Note:* In the field of urology, RLH has been reported in four cases involving the kidney, including this case.

Abbreviation: n.a., not available.

Radiologically, RLH can demonstrate enhancement patterns similar to those observed in malignant renal tumors. In the present case, early homogeneous enhancement of the renal lesion with similar enhancement of enlarged hilar lymph nodes strongly suggested metastatic renal cell carcinoma. However, significant lymphadenopathy in a small renal tumor with a diameter of 20 mm was atypical for malignant renal tumors.

Although the etiology of RLH remains unclear, potential associations with autoimmune diseases, chronic inflammation, and immune dysregulation have been proposed [10, 11]. While RLH generally follows a benign course, rare malignant transformation has been reported in other organs, emphasizing the importance of careful follow‐up [12, 13, 14, 15, 16].

In two previously reported cases of renal reactive lymphoid hyperplasia, the diagnosis was established by percutaneous renal biopsy. In one case, the preoperative diagnosis enabled renal‐sparing surgery, while in the other case, spontaneous regression of the lesion was observed during active surveillance.

Percutaneous renal biopsy has been reported to achieve a diagnostic accuracy exceeding 90%, and major complications, including clinically significant bleeding, occur in < 2% of cases, while tumor seeding is exceedingly rare (< 0.1%) [17, 18]. However, lesions located in close proximity to vital organs, major renal vessels, or the urinary collecting system, such as the pancreas, spleen, or bowel, may increase the risk of organ injury during percutaneous access. In such situations, interventional techniques, including CT‐guided hydrodissection or artificial pleural/peritoneal fluid instillation, have been described to create a safe biopsy window by separating adjacent structures [19].

In the present case, the tumor was located in close proximity to the pancreas. Although CT‐guided percutaneous biopsy could have been technically feasible, we considered that the potential risk of pancreatic injury outweighed the expected diagnostic benefit and therefore opted for surgical resection. Nevertheless, this case highlights that percutaneous biopsy should be actively considered in similar atypical presentations when a safe access route can be secured.

This report is limited by the relatively short follow‐up period of three months. Although the patient has remained recurrence‐free to date, longer‐term surveillance is warranted to definitively confirm the sustained benign clinical course and exclude the possibility of delayed progression.

In summary, although reactive lymphoid hyperplasia can be difficult to distinguish from renal cell carcinoma on contrast‐enhanced CT because of similar enhancement patterns, percutaneous needle biopsy should be actively considered when the clinical presentation is inconsistent with typical renal cell carcinoma, such as when the size of the renal tumor appears disproportionate to the presence of lymphadenopathy.

## 4 Conclusion

We report the first case of renal reactive lymphoid hyperplasia accompanied by regional lymphadenopathy. On contrast‐enhanced CT, the enhancement pattern closely resembled that of renal cell carcinoma. Reactive lymphoid hyperplasia should therefore be considered in the differential diagnosis of renal masses, and percutaneous needle biopsy should be actively considered when the clinical presentation is inconsistent with typical renal cell carcinoma.

## Ethics Statement

The authors have nothing to report.

## Consent

Written informed consent was obtained from the patient for publication of this case report and accompanying images.

## Conflicts of Interest

The authors declare no conflicts of interest.

## Data Availability

The data that support the findings of this study are available on request from the corresponding author. The data are not publicly available due to privacy or ethical restrictions.
